# Unbiased Functional Clustering of Gene Variants with a Phenotypic-Linkage Network

**DOI:** 10.1371/journal.pcbi.1003815

**Published:** 2014-08-28

**Authors:** Frantisek Honti, Stephen Meader, Caleb Webber

**Affiliations:** MRC Functional Genomics Unit, Department of Physiology, Anatomy and Genetics, University of Oxford, Oxford, United Kingdom; University of Toronto, Canada

## Abstract

Groupwise functional analysis of gene variants is becoming standard in next-generation sequencing studies. As the function of many genes is unknown and their classification to pathways is scant, functional associations between genes are often inferred from large-scale omics data. Such data types—including protein–protein interactions and gene co-expression networks—are used to examine the interrelations of the implicated genes. Statistical significance is assessed by comparing the interconnectedness of the mutated genes with that of random gene sets. However, interconnectedness can be affected by confounding bias, potentially resulting in false positive findings. We show that genes implicated through *de novo* sequence variants are biased in their coding-sequence length and longer genes tend to cluster together, which leads to exaggerated p-values in functional studies; we present here an integrative method that addresses these bias. To discern molecular pathways relevant to complex disease, we have inferred functional associations between human genes from diverse data types and assessed them with a novel phenotype-based method. Examining the functional association between *de novo* gene variants, we control for the heretofore unexplored confounding bias in coding-sequence length. We test different data types and networks and find that the disease-associated genes cluster more significantly in an integrated phenotypic-linkage network than in other gene networks. We present a tool of superior power to identify functional associations among genes mutated in the same disease even after accounting for significant sequencing study bias and demonstrate the suitability of this method to functionally cluster variant genes underlying polygenic disorders.

## Author Summary

Plenty of gene variants have been associated with a disease, yet most of the heritability, along with the molecular basis, of common diseases remains unexplained. However, it is widely thought that the products of genes whose mutations are implicated in the same disease function together in the same biological pathways and it is the disruption of these pathways that underlies the disease. Such pathways are not well defined and their identification could help elucidate disease mechanisms. Consequently, groupwise functional analyses of gene variants to identify common disease-relevant pathways are becoming standard in next-generation sequencing studies, but we find that these analyses are confounded by coding-sequence length bias. We control for these bias and describe a phenotype-based approach which outperforms other methods in discerning functional associations among the disease-associated genes. We also demonstrate the suitability of this method to functionally dissect the gene variants underlying a complex disorder, the identified functional clusters offering insight into disease mechanisms.

This is a *PLOS Computational Biology* Methods article.

## Introduction

It is widely postulated that the products of genes whose variants are implicated in the same disease participate in the same biological function or process whose disruption leads to the disease [1], [2]. This concept is supported by examples of complex disease in which the proteins encoded by the implicated genes interact, form a molecular complex or function at different steps of the same biochemical pathway [3], [4]. As there is limited power to associate rare variants with disease by case–control studies, the use of functional-enrichment approaches that identify a shared function in a set of mutated genes is becoming standard in the interpretation of variants [5]–[8].

Since the function of many genes is not known and their classification to pathways is scant, functional associations between genes are often inferred from large-scale omics data [4], [6]–[9]. However, the suitability of such data types, including protein–protein interactions and gene co-expression networks, for functional-enrichment analysis remains unclear. Moreover, the inferred functional associations can be affected by confounding factors, potentially resulting in false positive findings. Thus, it is important to identify any bias affecting the implicated genes and control for them. Multiple exome-sequencing studies currently test variants for functional enrichment and yet there is no consensus concerning what to control for [6]–[9].

In this study, we have inferred functional associations between human genes from diverse data types and assessed the phenotypic agreement of the inferred gene–gene associations. We have examined different data types and networks and found that genes mutated in the same disease cluster more significantly in an integrated phenotypic-linkage network than in other gene networks. Examining the functional association between *de novo* gene variants, we have identified a confounding bias in coding-sequence length that we control for. We present a tool that identifies functional associations among genes mutated in the same disease even after accounting for significant sequencing study bias and demonstrate the power of this tool to functionally subcluster the gene variants underlying a polygenic disorder.

## Results

**Figure 1:**
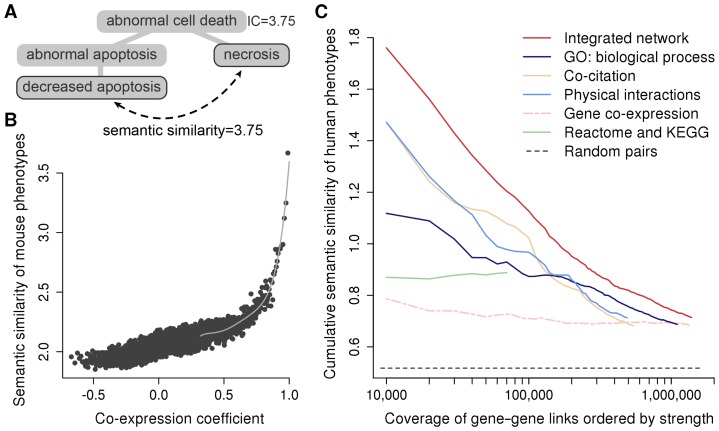
Processing and comparison of functional genomics data. (A) Terms in a phenotype ontology have an information content (IC) which is inversely proportional to the number of genes annotated with them. The semantic similarity between any two terms equals to the IC of their closest common ancestor term(s). (B) Gene–gene linkages derived from a data type are assessed and rescored according to the semantic similarity of the linked genes' mouse phenotype annotations. (C) The similarity in human phenotype annotations from the HPO is a benchmark on which all the data types can be compared, revealing their relative accuracy and coverage.

To test for functional associations among gene variants, we derived functional links between genes from diverse data types. For example, we calculated correlation coefficients from expression profiles, whereas gene annotation data were processed in the form of semantic similarity, which is a measure of relatedness between two genes assessed by the similarity of their annotations [10] (Figure 1A). The data were likely to include noise leading to false links and their reliability was unknown. To estimate and take into account the accuracy of the links, we evaluated the individual data types with a novel, phenotype-based method, by examining the semantic similarity between the mouse phenotypes of the genes they related to each other (Figure 1B). That is, each data type in turn indicated gene–gene linkages (gene pairs) and the accuracy of these links was assessed by considering the similarity of the phenotypes arising from the disruption of the unique mouse orthologs of these genes. We expected the data types to link together genes whose knockouts give rise to the same phenotypes, even if these mouse phenotypes were not necessarily expected to resemble human symptoms. The similarity of mouse phenotype annotations correlated with the similarity of human disease phenotypes (ρ = 0.223, P<2×10^−16^; Figure S1) and mouse phenotypes have been assigned to 6169 unique orthologs of human genes, 3.4-fold more than the 1801 genes annotated by the Human Phenotype Ontology (HPO; downloaded in 2012) [11]. Consequently, we used the phenotype annotations from the Mouse Genome Database [12] as the benchmark against which to evaluate other data types and set aside the HPO annotations for use as a test set for validation.

Integration of different data types into a combined network is expected to improve the accuracy of links and thus, in addition to considering individual data types, we also built an integrated gene network [13], [14]. For this, we selected data types that consistently linked together genes associated with similar mouse knockout phenotypes and that produced a positive correlation with the semantic similarity of mouse phenotypes (Table S1). For each data source suggesting functional links, we fitted regression curves in order to re-score the links so that any data-specific scores characterising the gene pairs were replaced with the semantic similarity that they corresponded to according to a regression function (see Figure 1B). When multiple data sources suggested functional linkage between the same two genes, we summed their link weights according to the approach of Marcotte and colleagues [15], thereby down-weighting less reliable data (see Methods). The resulting integrated gene network outperforms networks derived from the individual data types both in terms of coverage and accuracy (Figure 1C).

We corroborated the integrated phenotypic-linkage network by showing that genes whose perturbations are implicated in the same disease tend to be closely interlinked (Figures S2, S3, S4, S5, S6, S7). It is possible that their tendency to be closely interconnected is due to shared functional annotations assigned to them because they were implicated in the same disease in the literature. Also, we cannot assume that the associations of genes to phenotypes – forming the test sets – were made independently of any data type. Consequently, we turned to recently reported *de novo* mutations associated with developmental disorders that were identified independently of the data types included in the network.

**Figure 2:**
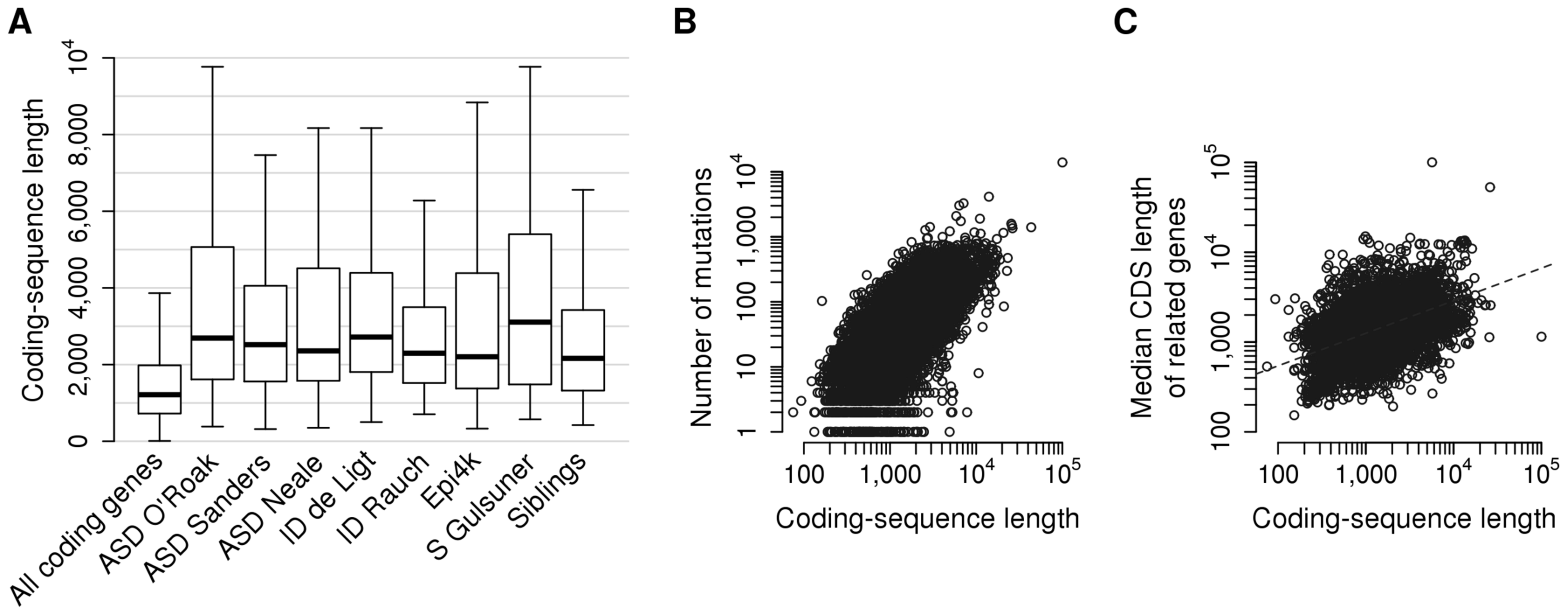
Coding sequence (CDS) lengths of genes with *de novo* variants. (A) ‘All genes’ denotes all translated human genes, ‘Siblings’ denotes genes with *de novo* mutations in non-autistic siblings of ASD cases published by O'Roak *et al.* and Sanders *et al.* Even the genes mutated in the healthy siblings are significantly longer than all coding genes (Mann–Whitney U test, P<2×10^−16^). The box plots depict the values between the 1^st^ and 3^rd^ quartile of a distribution, the 2^nd^ quartile (thick band) represents the median. (B) Mutational burden strongly correlates with coding sequence length in the Exome Variant Server (Spearman's ρ = 0.710, P<2×10^−16^; http://evs.gs.washington.edu/EVS). All nonsynonymous mutations were considered across all human chromosomes. (C) The median CDS length of a gene's connections correlates with its CDS length (Spearman's ρ = 0.508, P<2×10^−16^). We considered the strongest 100,000 links from the integrated phenotypic-linkage network.

**Figure 3:**
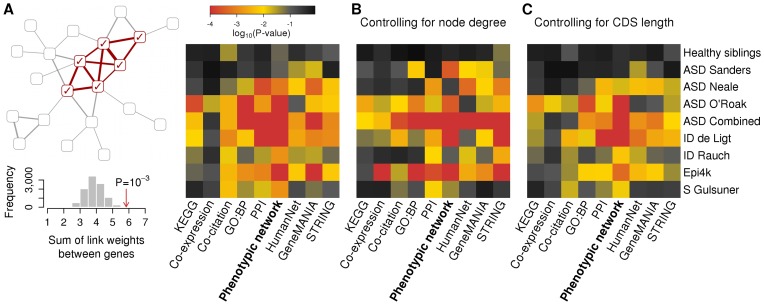
Clustering of genes hit by *de novo* nonsynonymous substitutions. (A) We have examined the network properties of whole sets of genes with nonsynonymous mutations implicated by recent exome-sequencing studies in autism (ASD), severe intellectual disability (ID), epilepsy or schizophrenia (S). We calculated the sum of link weights among genes from a set and compared this sum to that calculated for randomized gene sets in order to assess the degree of functional clustering. (B and C) The implicated genes are significantly more strongly interconnected with each other by means of functional genomics data than random gene sets of the same size, but controlling for coding sequence (CDS) length considerably affects the p-values. The genes mutated in the same disease cluster most significantly in the integrated phenotypic-linkage network, while genes mutated in healthy controls do not cluster.

Genes with *de novo* substitutions in patients with the same disorder [6]–[9], [16]–[18] showed a tendency to be more interconnected in the gene networks than random gene sets of the same size. However, as the interconnectedness of genes can be affected by confounding factors, it is important to identify any bias affecting the studied genes and control for them during the randomizations. We have found that the genes implicated through *de novo* sequence variants are biased in their coding-sequence (CDS) length, as longer genes are more likely to be mutated by chance (Figure 2). We also observe that genes with longer CDS tend to be interconnected (Figure 2C) and thus controlling for CDS length during the randomizations can significantly affect their relative degree of clustering (Figure 3). To control for coding-sequence (CDS) length during the randomizations, we have selected random genes the CDS length of which matched the CDS length of the studied candidate genes. Node degree has been previously identified as a confounding factor in functional analyses, particularly where an increase in degree results from study bias [19]. However, controlling for node degrees in a gene network does not correct the CDS length bias (Figure 3B). CDS length correlates very weakly with node degree (Spearman's ρ = 0.050). The length bias are highly significant in all the studied gene sets (Figure 2), while the node degrees are significantly different only in some of the candidate gene sets and there is no correlation between the node degree and mutational burden of genes (Figure S8). Having examined different data types and networks [15], [20], [21], we find that the disease-associated genes cluster more significantly in the integrated phenotypic-linkage network than in other gene networks (Figure 3).

## Discussion

We have inferred functional-association networks of human genes from diverse data types and assessed the phenotypic agreement of the inferred links. Having examined different data types and networks, we have found that genes mutated in the same disease cluster more significantly in an integrated phenotypic-linkage network than in other gene networks (Figure 3C). We note that another gene network, called NETBAG, has been developed by Gilman and colleagues [22]. We could not access NETBAG for the performance comparison. Nevertheless, Gilman and colleagues state the use of shared disease associations among 478 human genes as the gold standard in their network construction [22] and the used disease associations originate from a study published in 2001 [23]. By comparison, our method takes advantage of over 100,000 mammalian genotype–phenotype relations and fully exploits bio-ontologies by means of semantic similarity, with both advances expected to enhance greatly the phenotypic-linkage network that we explicitly present here.

Examining the functional association between *de novo* gene variants, we have identified a confounding bias in coding-sequence length that we control for to avoid false positive findings. Numerous implicated variants are in fact expected to be neutral mutations but they are more likely to appear in genes with longer CDS, leading to a tendency of the implicated genes to be interconnected in gene networks (see Figure 2). These bias have confounded functional analyses and likely led to an overestimation of functional clustering in former studies. We have found that the CDS-length bias were highly significant in all the studied gene sets, including the unaffected siblings, while the node degrees were not. The higher node degrees in some of the candidate gene sets may indicate a functional signal, as the same genes are significantly more conserved (Figure S8). We conclude that controlling for CDS length in functional analyses of gene variants is appropriate.

One way of controlling for CDS length is to compare the interconnectedness of the implicated genes with that of genes mutated in unaffected controls [9]. However, we observe that the control genes tend to be less interconnected than random genes (Figure S9), which suggests that our way of controlling for CDS length (see Methods) is more conservative.

The nature of the phenotypic-linkage network suggests that the clustered genes function together in the same disease-relevant cellular pathways (Figure S10). The functional convergence that we identify among the three sets of genes from independent exome studies of autism spectrum disorder demonstrates that the method is able to detect biological coherence among variant genes (Figures 3 and S10). Throughout, we have considered the larger class of non-synonymous variants which is likely to possess a more diluted signal than nonsense variants. As with all clustering methods, our method is sensitive to the number of variants identified and the likelihood of their causal relation. Half of our study sets included only 5–10 genes with nonsense variants, between which we either did not find any functional links or the sum of link weights was not significantly higher than expected after controlling for CDS length. For studies of rare or *de novo* variants derived from a single or small number of genomes, gene prioritizing methods based upon phenotypic similarity may be more appropriate [24]. Continuing efforts to systematically phenotype model organisms and to enrich the phenotype ontologies could further improve the resulting phenotypic-linkage networks that are constructed [25]. The integrated network toolkit is made available at http://groups.mrcfgu.ox.ac.uk/webber-group/resources.

## Methods

### Inference of functional associations between genes from diverse data types

To gain the most information about genes whose variants may be relevant to disease and to explore the functional relations between them, we collected large amounts of functional genomics data on human genes and their mouse orthologs. We wanted the data sets to inform us about the functional similarity of genes, therefore we processed them such that they indicated gene–gene links. For every data type except physical interactions, we derived a score characterising gene pairs, such as the correlation coefficient from expression profiles or semantic similarity from gene annotations.

### Semantic similarity

All gene annotation data (such as GO, KEGG, Reactome, InterPro and mouse and human phenotype annotations) were processed in the form of semantic similarity, which is a measure of relatedness between two genes assessed by the similarity of their annotations [10]. The terms used to annotate genes have an information content (IC) defined as:
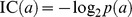
where *p*(*a*) is the proportion of genes annotated with term *a* or its descendent terms among all genes with an annotation.

We used Resnik's [26] measure together with the GraSM approach [27] to calculate the similarity of terms organized in a hierarchical ontology, defining the semantic similarity between any two terms *t*_1_ and *t*_2_ as the average IC of their disjunct common ancestor terms (see Figure 1A):
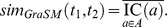



To measure the functional relatedness of two genes, we compared their annotations with the maximum (max) and best match average (bma) methods [28]. Let *T*_1_ denote the set of terms annotated to gene *g*_1_and *T*_2_ denote the set of terms annotated to gene *g*_2_, the semantic similarity of their annotations according to the max approach is then given by:
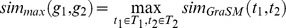
while the semantic similarity of their annotations according to the bma approach is defined as:
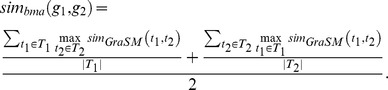



### Data sources

#### Gene expression

We inferred gene–gene linkages from co-expression of genes. To measure the co-expression of genes, we calculated the Pearson's correlation coefficient of their expression profiles, requiring at least ten tissues in which both genes were expressed for the calculation of a correlation coefficient. We used expression data from GNF2 [29], GSE3594 [30], MTAB-62 [31] and further five sets [32]–[36], calculating the Pearson's correlation coefficients within each, evaluating the inferred gene–gene links as described below (see Figure 1B), selecting and re-scoring the links that correlated with the semantic similarity of mouse phenotypes and integrating these links as described below to create a combined co-expression network. The resulting integrated network outperformed networks derived from the individual co-expression datasets both in terms of coverage and accuracy.

#### Physical interactions

Protein–protein interactions provided a binary measure of functional linkage between genes, with all derived gene pairs receiving the same score. We measured the median semantic similarity of mouse phenotype annotations for all the gene pairs derived from the same assay and used this median value to score all the functional linkages in the given data set. We used physical interaction data divided by assay types from BioGRID [37] v3.1.72, IntAct [38] (downloaded on July 29, 2011), CORUM [39] (downloaded on May 5, 2011), DICS [40] (downloaded on June 6, 2011) and Reactome [41] (downloaded on May 5, 2011). We also derived indirect links based on shared interaction partners and accorded with their own weights and integrated these with the direct links to create a combined PPI network.

#### Co-citation

Co-citation scores were extracted from STRING [21] v8.3. We used the co-citation scores of mouse orthologs of human genes.

#### Gene annotations

Gene annotations were obtained from the Gene Ontology [42] (GO, downloaded on July 29, 2011), using the annotations to human and mouse genes in the biological process (BP), molecular function and cellular location categories, with evidence codes IDA, IMP, TAS and IC. Pathway annotations of mouse genes were obtained from KEGG [43] (downloaded on March 30, 2011), pathway annotations of human genes were downloaded from Reactome [41] (on March 23, 2011). Protein domain annotations were obtained from InterPro [44] (downloaded on May 23, 2011). Mouse phenotype annotations were obtained from the Mouse Genome Database [12] (downloaded on August 24, 2011), human phenotype annotations were downloaded from the Human Phenotype Ontology [11] (on August 8, 2012). The annotation terms in KEGG, Reactome and InterPro are not organized in a deep ontology with many levels, therefore we only used direct matches between these gene annotations with the maximum (max) method in calculating semantic similarity scores for gene pairs.

### Evaluation of data sets

To estimate the reliability of the individual data sets, we evaluated them by examining the semantic similarity between the phenotypes associated with the unique mouse orthologs of the genes they linked to each other. For each data set, we derived gene–gene linkages (gene pairs) with data-specific scores characterizing the strength of a linkage and ordered the gene pairs by their score from largest to smallest. Next, we calculated and plotted the median semantic similarity of mouse phenotype annotations for bins of 1,000 gene pairs (see Figure 1B).

We tested if the data types linked together genes whose knockouts influence the same phenotypes. When the strongest linkages derived from a data set did not correspond to higher semantic similarities of phenotypes than expected by chance, we did not include the links from the given set in the integrated gene network (Table S2).

### Re-scoring and integration of data

We selected data sets that produced a positive correlation with the semantic similarity of mouse phenotypes (Table S1) and fitted regression curves in order to re-score the links so that any data-specific scores characterising the gene pairs were replaced with the semantic similarity of phenotypes that they corresponded to according to a linear regression function (Figure 1B). Thus all gene pairs that had an original data-specific score were re-scored, including those that did not have phenotypic annotations.

By re-scoring the data types with a universal benchmark we weighted them in proportion of their relative accuracy. When multiple data sources suggested functional linkage between the same two genes, we summed their link weights (Figure S11) increasingly down-weighting less reliable data according to a formula used by Marcotte and colleagues [15]:
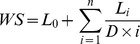
where *L* represents a re-scored link weight from a single data set, *L*_0_ being the largest link weight among all the links between the given two genes, *i* is the index of the remaining links ordered by their weights for the gene pair and *D* is a free parameter. We optimized the value of this parameter and used *D* = 5 in integrating data types to create the final phenotypic-linkage network.

### Controlling for coding-sequence length

In testing for functional enrichment in a set of genes, the degree of functional association between the genes can be compared to that calculated for randomized gene sets. As the degree of functional association can be affected by confounding bias, it is important to identify such bias affecting the studied gene set and control for them. To control for coding-sequence (CDS) length during the randomizations, we selected random genes the CDS length of which matched the CDS length of the studied (mutated) genes. For each of the studied genes in turn we assigned a list of 100 genes with the same or most similar CDS length, using the longest CDS of each gene. Random gene sets were then assembled by selecting one random gene from each of these lists.

## Supporting Information

Figure S1 **Correlation between semantic similarities measured with different gene annotations.** Gene pairs were ordered by their semantic similarity scores based on either the human Gene Ontology biological process (grey) or mouse phenotype annotations to genes (black dots). The ordered pairs were divided to bins of 1,000 and the median of the semantic similarity scores measured with Human Phenotype Ontology annotations has been calculated for each bin of gene pairs.
(PNG)

Figure S2 **Clustering of genes for Human Phenotype Ontology (HPO) phenotypes in a gene network built on the semantic similarity of mouse phenotypes.** We calculated the sum of link weights among genes annotated with the same symptom and used it to represent the degree of clustering of these sets of genes. The box plots show the distribution of the sums of link weights for 100,000 sets of randomly selected genes with the same node degrees as the seed genes. The sums of link weights are presented as fold changes compared to the median of the specific distribution, set to equal 1 for each term. For each HPO phenotype, we randomly selected the same number of genes as there were annotated with that symptom in the HPO. This number is shown in parentheses; the red marks indicate the sum of link weights among the actual genes annotated with the corresponding HPO term.
(PNG)

Figure S3 **Clustering of genes for Human Phenotype Ontology (HPO) phenotypes in a gene network built on the semantic similarity of Gene Ontology biological process annotations.** We calculated the sum of link weights among genes annotated with the same symptom and used it to represent the degree of clustering of these sets of genes. The box plots show the distribution of the sums of link weights for 100,000 sets of randomly selected genes with the same node degrees as the seed genes. The sums of link weights are presented as fold changes compared to the median of the specific distribution, set to equal 1 for each term. For each HPO phenotype, we randomly selected the same number of genes as there were annotated with that symptom in the HPO. This number is shown in parentheses; the red marks indicate the sum of link weights among the actual genes annotated with the corresponding HPO term.
(PNG)

Figure S4 **Clustering of genes for Human Phenotype Ontology (HPO) phenotypes in a gene network based on protein–protein interactions.** We calculated the sum of link weights among genes annotated with the same symptom and used it to represent the degree of clustering of these sets of genes. The box plots show the distribution of the sums of link weights for 100,000 sets of randomly selected genes with the same node degrees as the seed genes. The sums of link weights are presented as fold changes compared to the median of the specific distribution, set to equal 1 for each term. For each HPO phenotype, we randomly selected the same number of genes as there were annotated with that symptom in the HPO. This number is shown in parentheses; the red marks indicate the sum of link weights among the actual genes annotated with the corresponding HPO term.
(PNG)

Figure S5 **Clustering of genes for Human Phenotype Ontology (HPO) phenotypes in a gene network built on the co-citation of mouse genes.** We calculated the sum of link weights among genes annotated with the same symptom and used it to represent the degree of clustering of these sets of genes. The box plots show the distribution of the sums of link weights for 100,000 sets of randomly selected genes with the same node degrees as the seed genes. The sums of link weights are presented as fold changes compared to the median of the specific distribution, set to equal 1 for each term. For each HPO phenotype, we randomly selected the same number of genes as there were annotated with that symptom in the HPO. This number is shown in parentheses; the red marks indicate the sum of link weights among the actual genes annotated with the corresponding HPO term.
(PNG)

Figure S6 **Clustering of genes for Human Phenotype Ontology (HPO) phenotypes in an integrated co-expression network based on microarrays.** We calculated the sum of link weights among genes annotated with the same symptom and used it to represent the degree of clustering of these sets of genes. The box plots show the distribution of the sums of link weights for 100,000 sets of randomly selected genes with the same node degrees as the seed genes. The sums of link weights are presented as fold changes compared to the median of the specific distribution, set to equal 1 for each term. For each HPO phenotype, we randomly selected the same number of genes as there were annotated with that symptom in the HPO. This number is shown in parentheses; the red marks indicate the sum of link weights among the actual genes annotated with the corresponding HPO term.
(PNG)

Figure S7 **Clustering of genes for Human Phenotype Ontology (HPO) phenotypes in the integrated phenotypic-linkage network.** We calculated the sum of link weights among genes annotated with the same symptom and used it to represent the degree of clustering of these sets of genes. The box plots show the distribution of the sums of link weights for 100,000 sets of randomly selected genes with the same node degrees as the seed genes. The sums of link weights are presented as fold changes compared to the median of the specific distribution, set to equal 1 for each term. For each HPO phenotype, we randomly selected the same number of genes as there were annotated with that symptom in the HPO. This number is shown in parentheses; the red marks indicate the sum of link weights among the actual genes annotated with the corresponding HPO term.
(PNG)

Figure S8 **Node degrees of genes with** ***de novo*** **variants.** (A) ‘All EVS genes’ denotes all genes in the Exome Variant Server, ‘Siblings’ denotes genes with *de novo* mutations in non-autistic siblings of ASD cases published by O'Roak *et al.* and Sanders *et al.* The node degrees are significantly higher only in the O'Roak *et al.*, de Ligt *et al.*, Rauch *et al.* and Epi4k candidate gene sets. The node degrees represent the number of connections of a gene in the integrated phenotypic-linkage network. (B) Mutational burden does not correlate with node degree in the Exome Variant Server (Spearman's ρ = 0.007; http://evs.gs.washington.edu/EVS). All nonsynonymous mutations were considered across all human chromosomes. (C) The same gene sets that have higher node degrees show significantly increased sequence conservation (lower K_a_/K_s_ ratio), indicating that the degree bias could be due to a functional signal in these gene sets. K_a_/K_s_ is the ratio of the number of nonsynonymous substitutions per nonsynonymous site (K_a_) to the number of synonymous substitutions per synonymous site (K_s_), based on one-to-one orthologs between human and mouse genes.
(TIF)

Figure S9 **Interconnectedness of controls used in simulations.** We calculated the number of links between 54 randomly selected control genes carrying damaging mutations in unaffected siblings, as in Gulsuner *et al.*, in the GeneMania physical interaction data set (http://pages.genemania.org/data). We also calculated the number of links between randomly selected genes matched in CDS length to the genes mutated in the Gulsuner *et al.* probands in the same network (*Random genes*). The box plots show the distribution of the numbers of links for 10,000 sets of randomly selected genes. The null distribution used in controlling for CDS length has a larger spread, indicating that controlling for CDS length in testing for clustering is more conservative.
(PNG)

Figure S10 **Functional subclusters of genes implicated in autism within the integrated gene network.** Only the strongest 166 links are shown among 115 genes. The terms represent the most significantly enriched GO biological process annotations among the genes forming the subclusters. Links based on the semantic similarity of GO annotations were included in the integrated network, but these enrichments are still useful in characterizing the subclusters and illustrate that the subclusters fit well with recent insights into the etiological variation underlying ASD [45].
(PNG)

Figure S11 **Integration of different data types linking genes.** When multiple data sources suggested functional linkage between the same two genes, we integrated the link weights into one for each gene pair. The rounded rectangles represent genes.
(PNG)

Table S1 **Data sources included in the integrated phenotypic-linkage network.**
(DOC)

Table S2 **Data sources not included in the integrated phenotypic-linkage network.**
(DOC)

Table S3 **Fractions of gene pairs co-annotated with the same phenotype in the integrated phenotypic-linkage network.**
(DOC)
