# Supplementary figures and images for: Unbiased Functional Clustering of Gene Variants with a Phenotypic-Linkage Network
Source: PLoS Comput Biol. 2014 Aug 28;10(8):e1003815. doi: 10.1371/journal.pcbi.1003815 (PMC4148192; doi:10.1371/journal.pcbi.1003815)

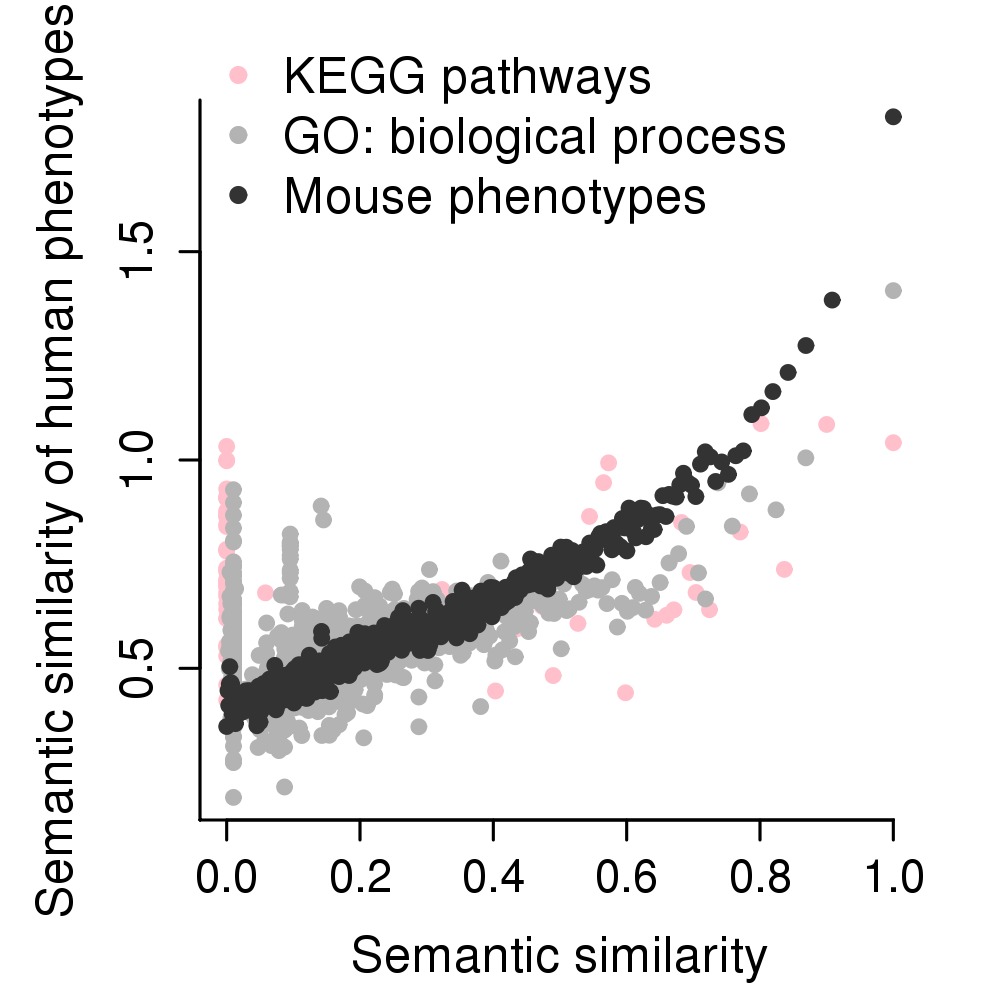

Supplement: Figure S1 — Correlation between semantic similarities measured with different gene annotations. Gene pairs were ordered by their semantic similarity scores based on either the human Gene Ontology biological process (grey) or mouse phenotype annotations to genes (black dots). The ordered pairs were divided to bins of 1,000 and the median of the semantic similarity scores measured with Human Phenotype Ontology annotations has been calculated for each bin of gene pairs. (PNG) [file pcbi.1003815.s001.png]

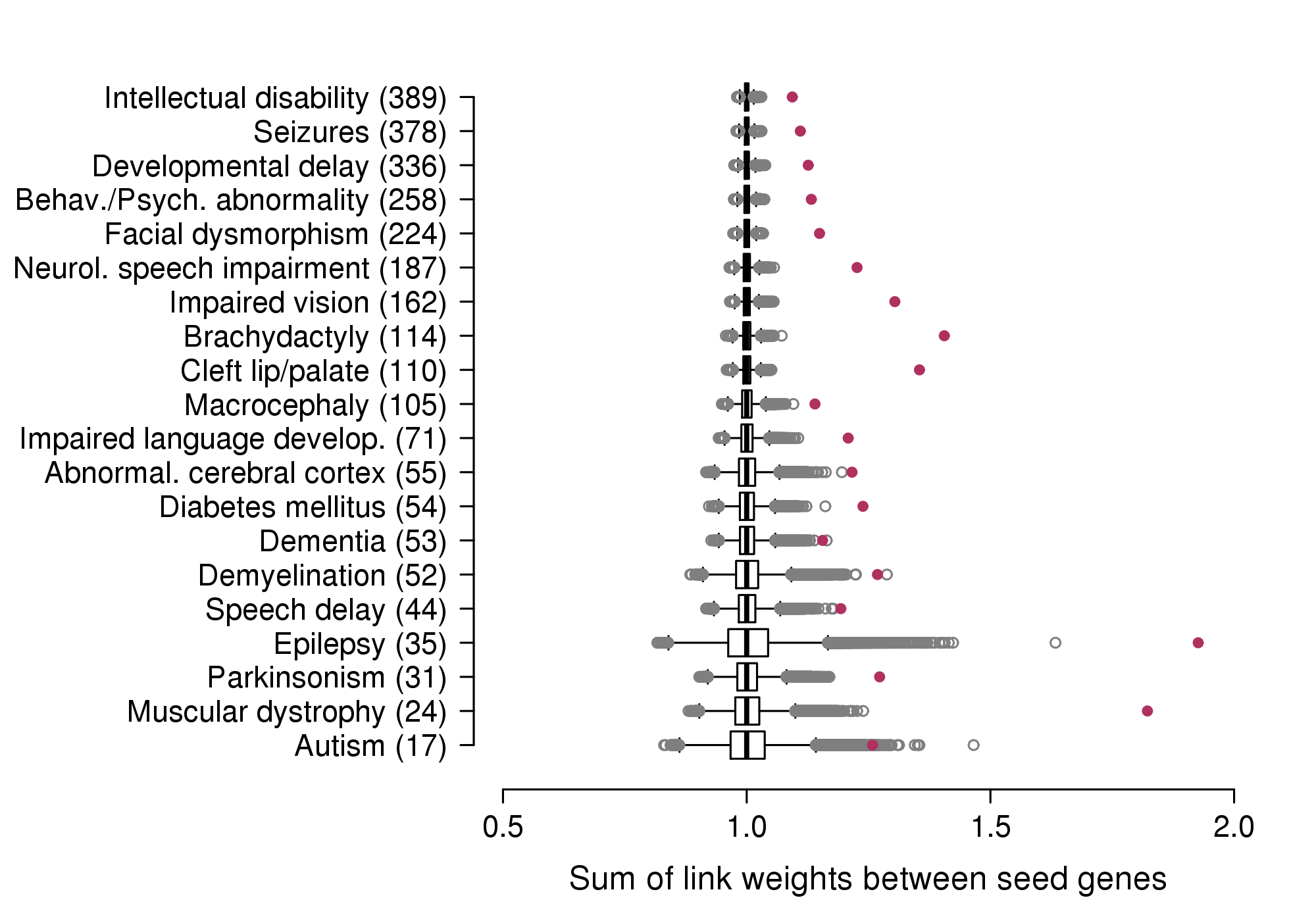

Supplement: Figure S2 — Clustering of genes for Human Phenotype Ontology (HPO) phenotypes in a gene network built on the semantic similarity of mouse phenotypes. We calculated the sum of link weights among genes annotated with the same symptom and used it to represent the degree of clustering of these sets of genes. The box plots show the distribution of the sums of link weights for 100,000 sets of randomly selected genes with the same node degrees as the seed genes. The sums of link weights are presented as fold changes compared to the median of the specific distribution, set to equal 1 for each term. For each HPO phenotype, we randomly selected the same number of genes as there were annotated with that symptom in the HPO. This number is shown in parentheses; the red marks indicate the sum of link weights among the actual genes annotated with the corresponding HPO term. (PNG) [file pcbi.1003815.s002.png]

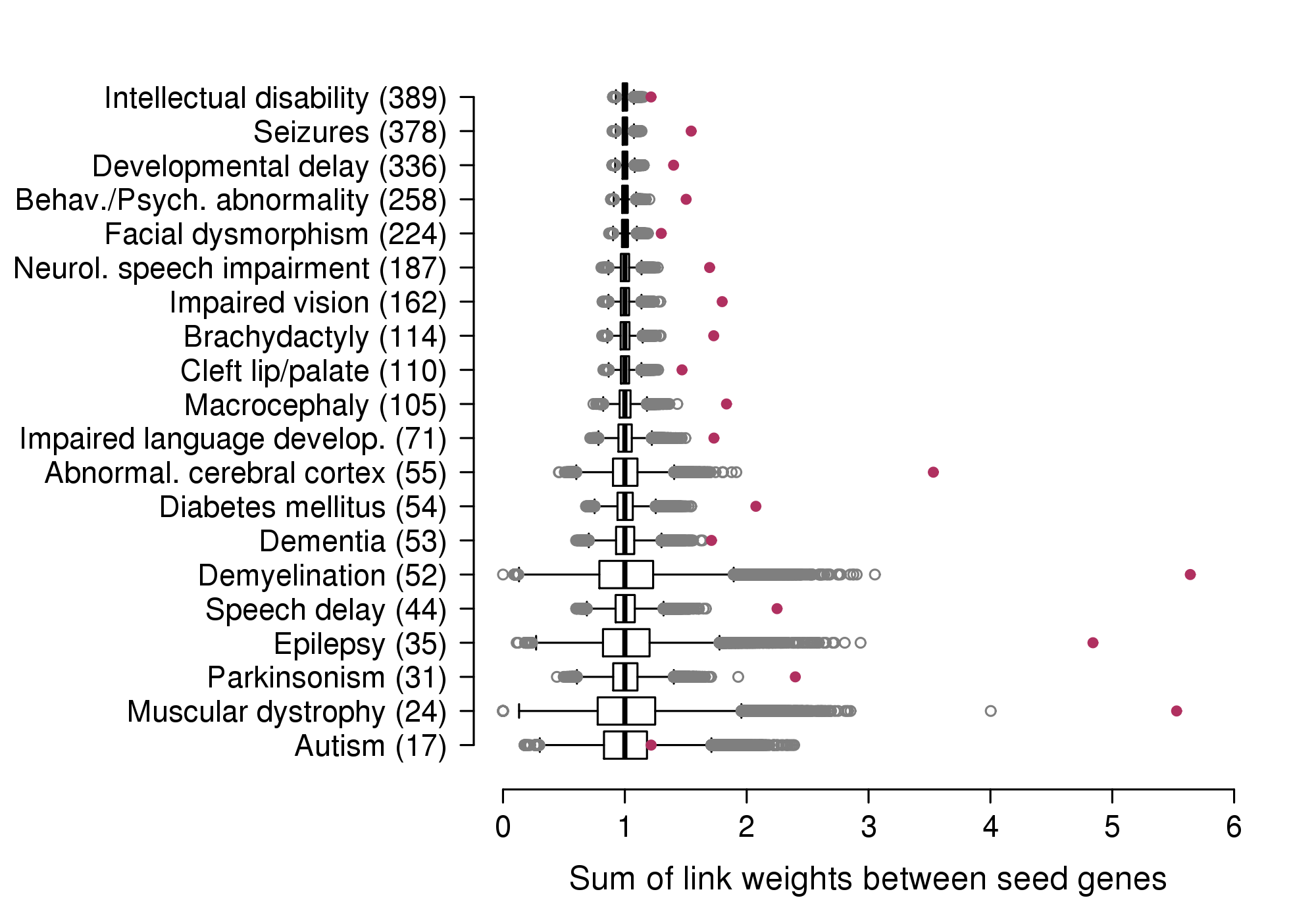

Supplement: Figure S3 — Clustering of genes for Human Phenotype Ontology (HPO) phenotypes in a gene network built on the semantic similarity of Gene Ontology biological process annotations. We calculated the sum of link weights among genes annotated with the same symptom and used it to represent the degree of clustering of these sets of genes. The box plots show the distribution of the sums of link weights for 100,000 sets of randomly selected genes with the same node degrees as the seed genes. The sums of link weights are presented as fold changes compared to the median of the specific distribution, set to equal 1 for each term. For each HPO phenotype, we randomly selected the same number of genes as there were annotated with that symptom in the HPO. This number is shown in parentheses; the red marks indicate the sum of link weights among the actual genes annotated with the corresponding HPO term. (PNG) [file pcbi.1003815.s003.png]

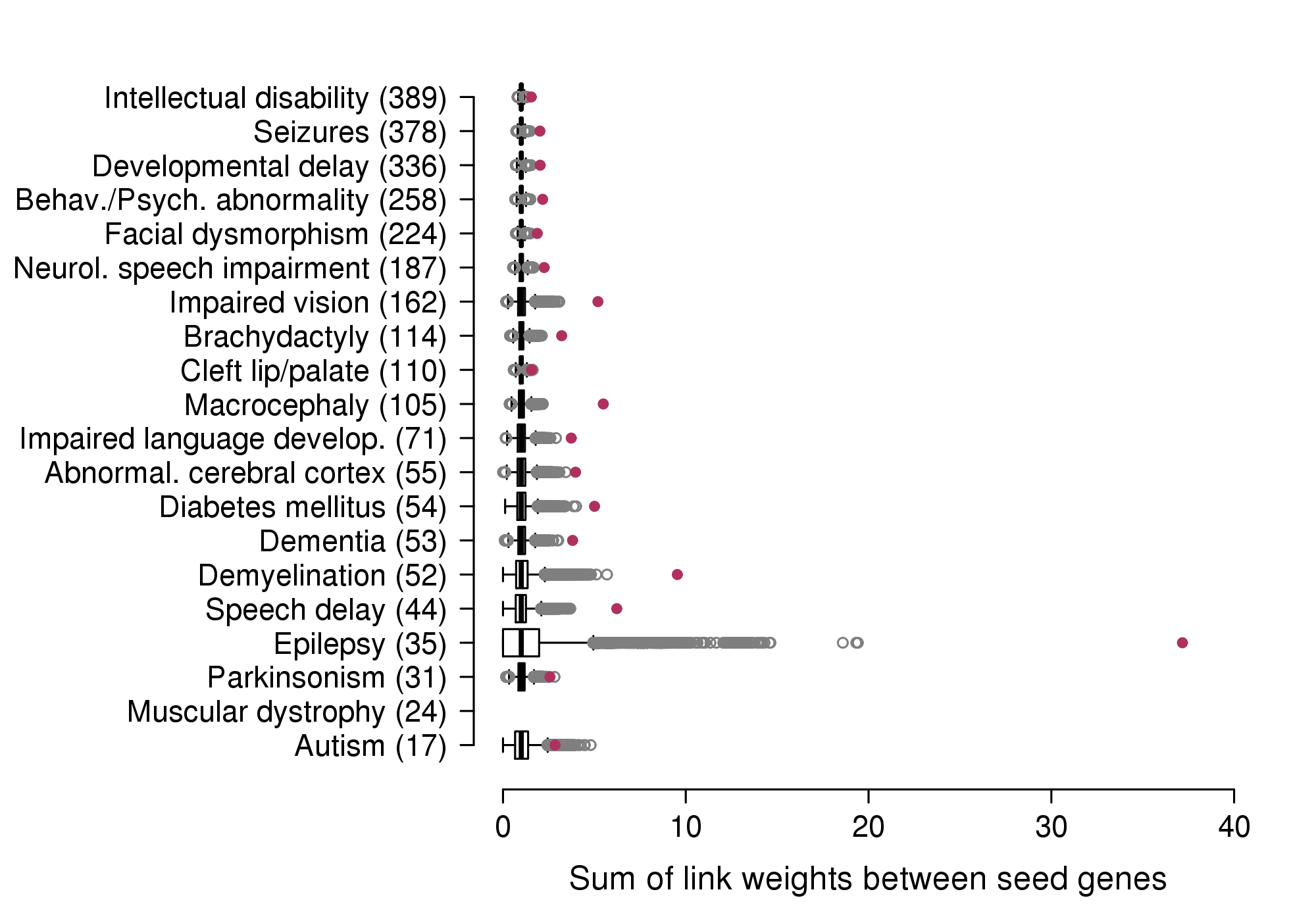

Supplement: Figure S4 — Clustering of genes for Human Phenotype Ontology (HPO) phenotypes in a gene network based on protein–protein interactions. We calculated the sum of link weights among genes annotated with the same symptom and used it to represent the degree of clustering of these sets of genes. The box plots show the distribution of the sums of link weights for 100,000 sets of randomly selected genes with the same node degrees as the seed genes. The sums of link weights are presented as fold changes compared to the median of the specific distribution, set to equal 1 for each term. For each HPO phenotype, we randomly selected the same number of genes as there were annotated with that symptom in the HPO. This number is shown in parentheses; the red marks indicate the sum of link weights among the actual genes annotated with the corresponding HPO term. (PNG) [file pcbi.1003815.s004.png]

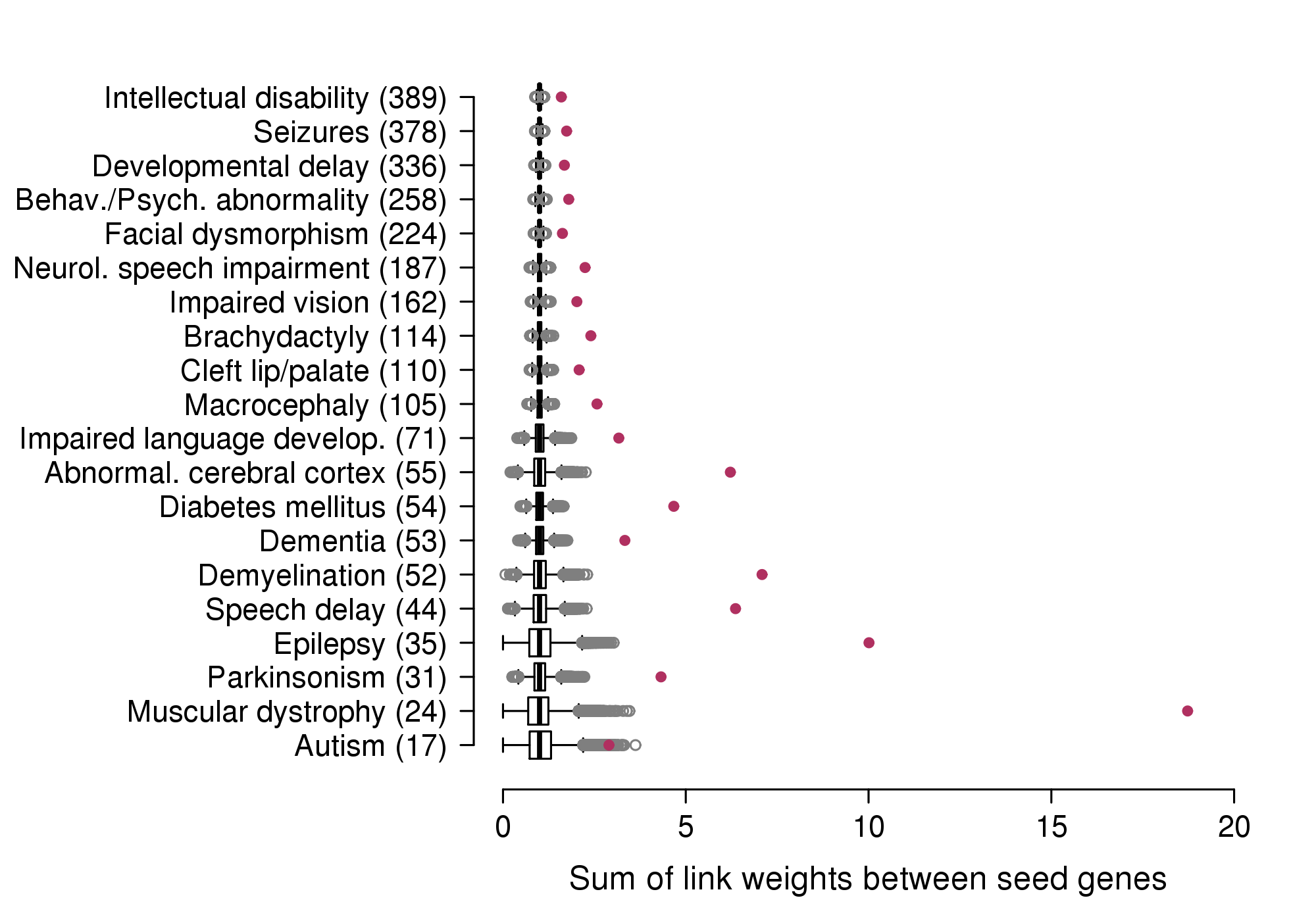

Supplement: Figure S5 — Clustering of genes for Human Phenotype Ontology (HPO) phenotypes in a gene network built on the co-citation of mouse genes. We calculated the sum of link weights among genes annotated with the same symptom and used it to represent the degree of clustering of these sets of genes. The box plots show the distribution of the sums of link weights for 100,000 sets of randomly selected genes with the same node degrees as the seed genes. The sums of link weights are presented as fold changes compared to the median of the specific distribution, set to equal 1 for each term. For each HPO phenotype, we randomly selected the same number of genes as there were annotated with that symptom in the HPO. This number is shown in parentheses; the red marks indicate the sum of link weights among the actual genes annotated with the corresponding HPO term. (PNG) [file pcbi.1003815.s005.png]

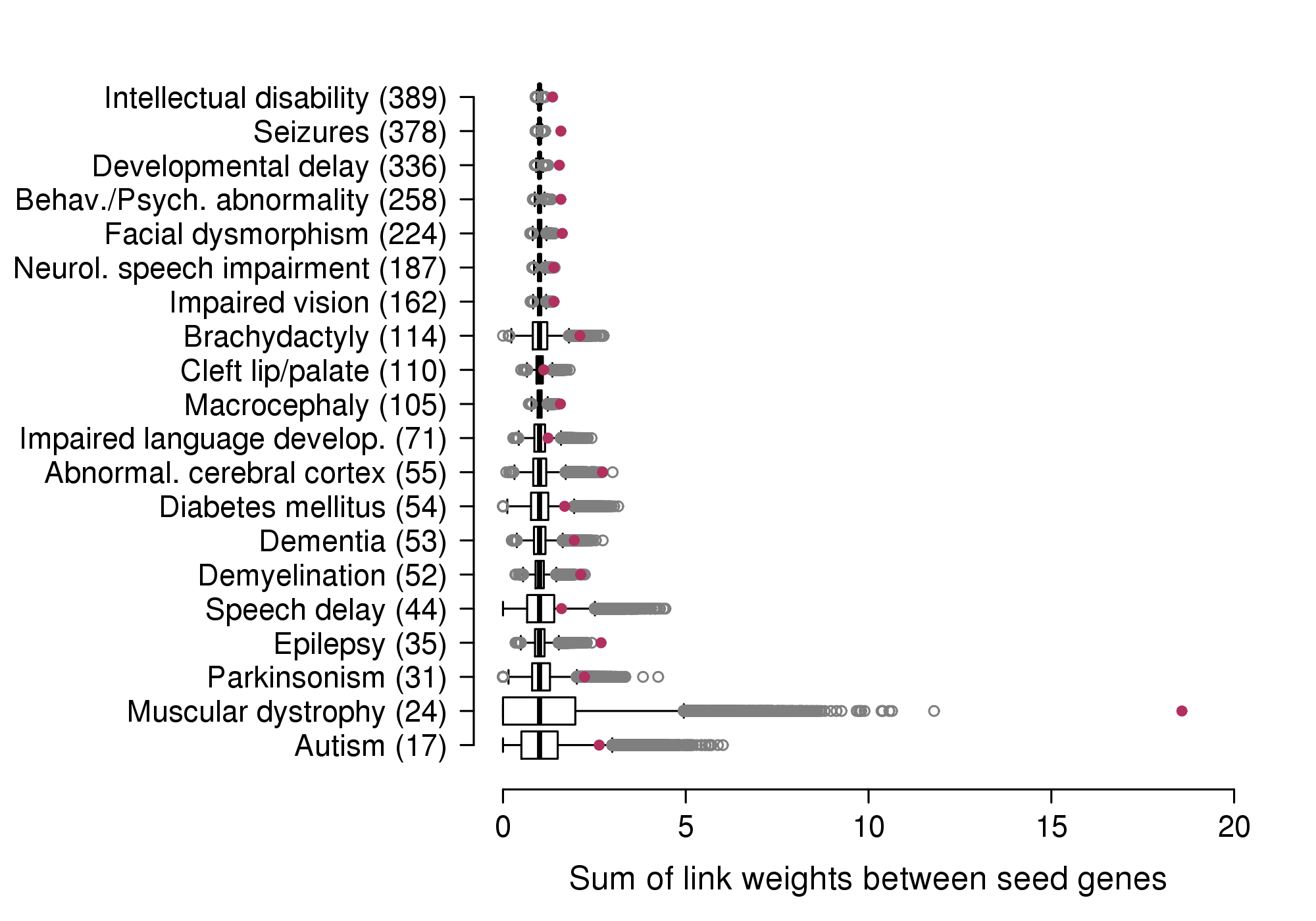

Supplement: Figure S6 — Clustering of genes for Human Phenotype Ontology (HPO) phenotypes in an integrated co-expression network based on microarrays. We calculated the sum of link weights among genes annotated with the same symptom and used it to represent the degree of clustering of these sets of genes. The box plots show the distribution of the sums of link weights for 100,000 sets of randomly selected genes with the same node degrees as the seed genes. The sums of link weights are presented as fold changes compared to the median of the specific distribution, set to equal 1 for each term. For each HPO phenotype, we randomly selected the same number of genes as there were annotated with that symptom in the HPO. This number is shown in parentheses; the red marks indicate the sum of link weights among the actual genes annotated with the corresponding HPO term. (PNG) [file pcbi.1003815.s006.png]

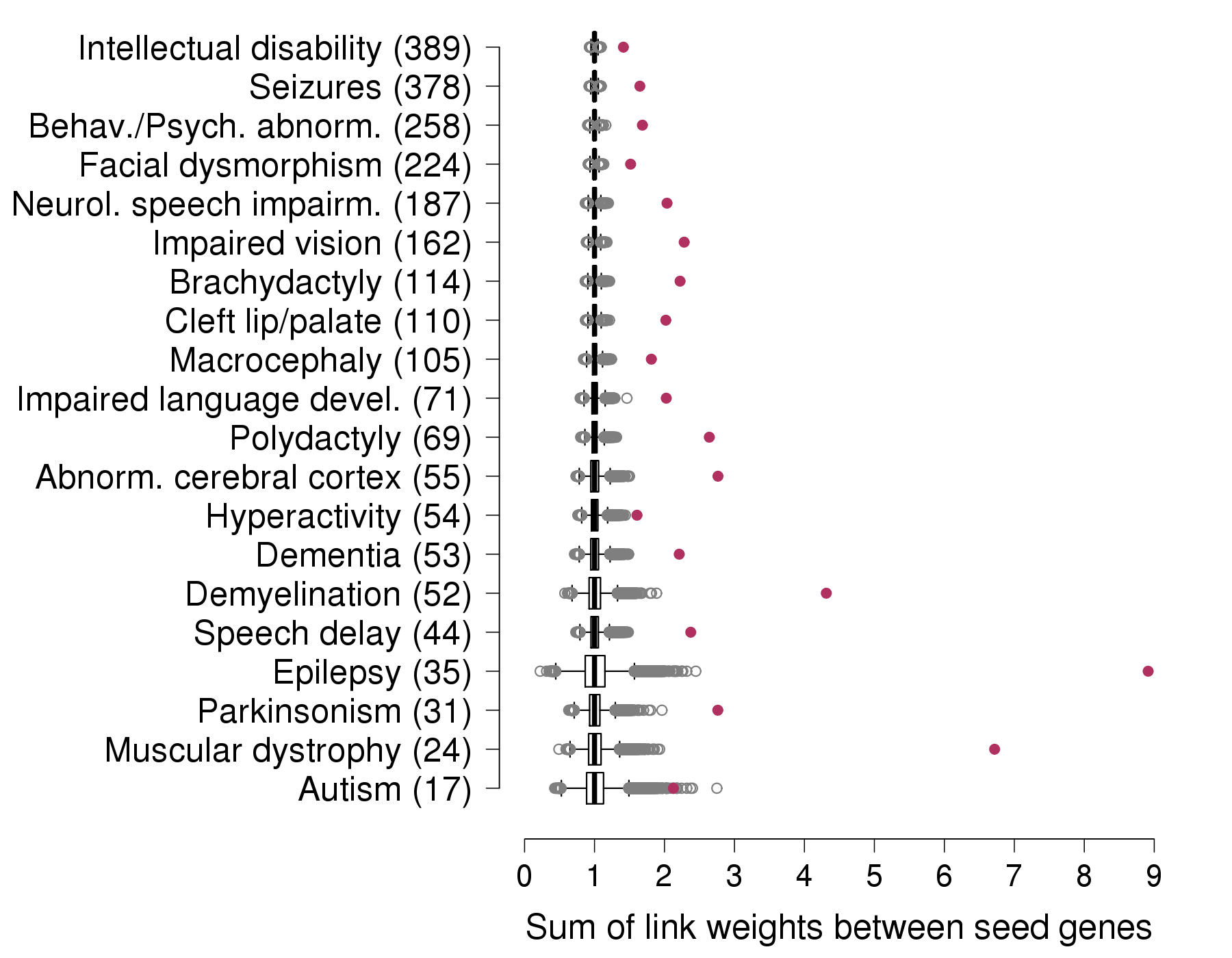

Supplement: Figure S7 — Clustering of genes for Human Phenotype Ontology (HPO) phenotypes in the integrated phenotypic-linkage network. We calculated the sum of link weights among genes annotated with the same symptom and used it to represent the degree of clustering of these sets of genes. The box plots show the distribution of the sums of link weights for 100,000 sets of randomly selected genes with the same node degrees as the seed genes. The sums of link weights are presented as fold changes compared to the median of the specific distribution, set to equal 1 for each term. For each HPO phenotype, we randomly selected the same number of genes as there were annotated with that symptom in the HPO. This number is shown in parentheses; the red marks indicate the sum of link weights among the actual genes annotated with the corresponding HPO term. (PNG) [file pcbi.1003815.s007.png]

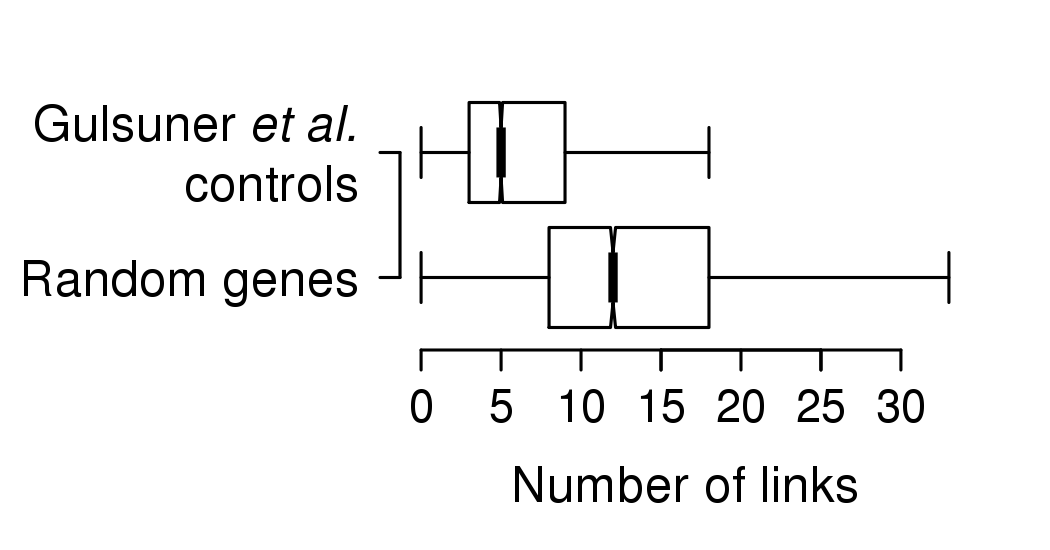

Supplement: Figure S9 — Interconnectedness of controls used in simulations. We calculated the number of links between 54 randomly selected control genes carrying damaging mutations in unaffected siblings, as in Gulsuner et al., in the GeneMania physical interaction data set (http://pages.genemania.org/data). We also calculated the number of links between randomly selected genes matched in CDS length to the genes mutated in the Gulsuner et al. probands in the same network (Random genes). The box plots show the distribution of the numbers of links for 10,000 sets of randomly selected genes. The null distribution used in controlling for CDS length has a larger spread, indicating that controlling for CDS length in testing for clustering is more conservative. (PNG) [file pcbi.1003815.s009.png]

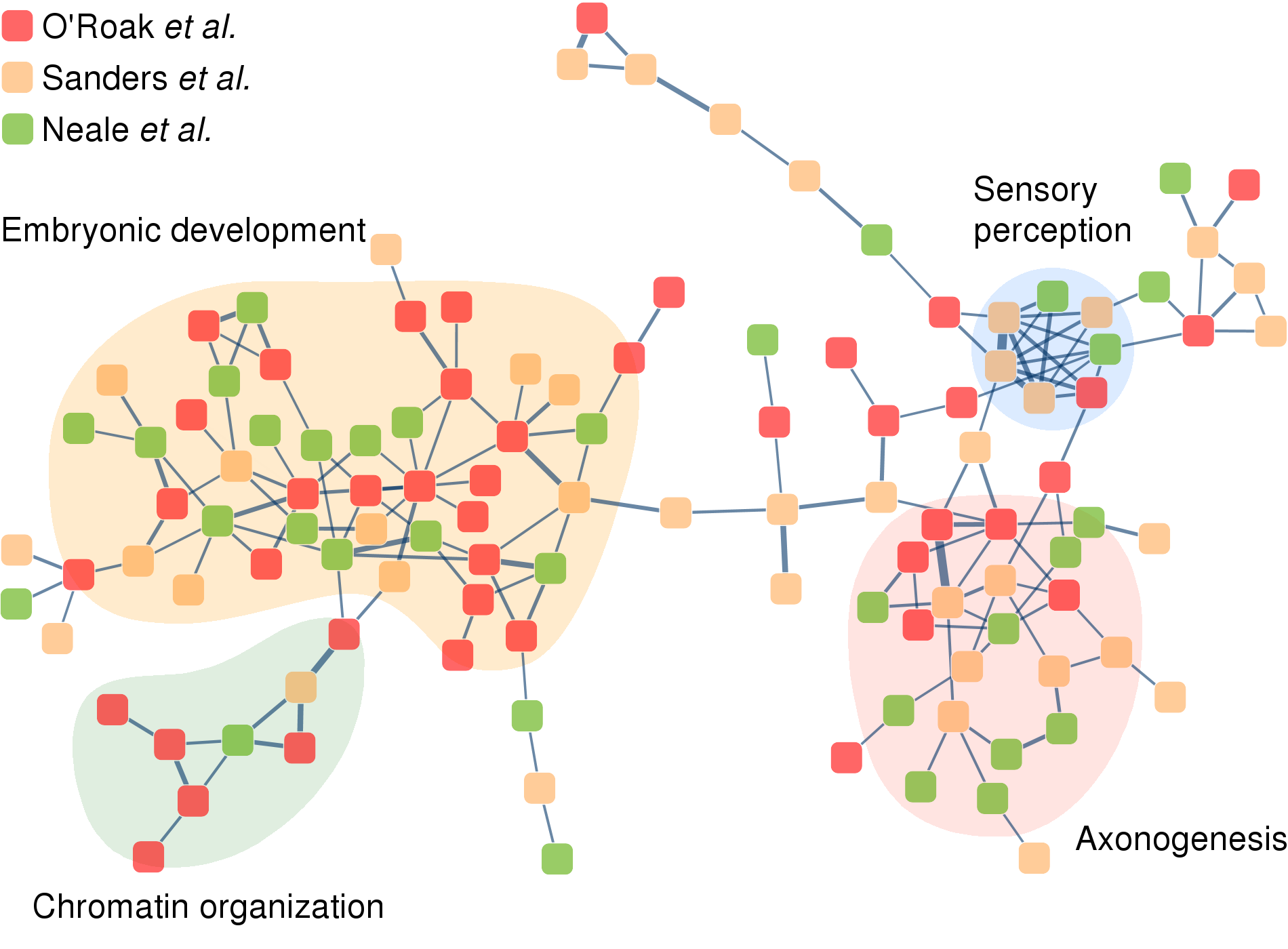

Supplement: Figure S10 — Functional subclusters of genes implicated in autism within the integrated gene network. Only the strongest 166 links are shown among 115 genes. The terms represent the most significantly enriched GO biological process annotations among the genes forming the subclusters. Links based on the semantic similarity of GO annotations were included in the integrated network, but these enrichments are still useful in characterizing the subclusters and illustrate that the subclusters fit well with recent insights into the etiological variation underlying ASD [45]. (PNG) [file pcbi.1003815.s010.png]

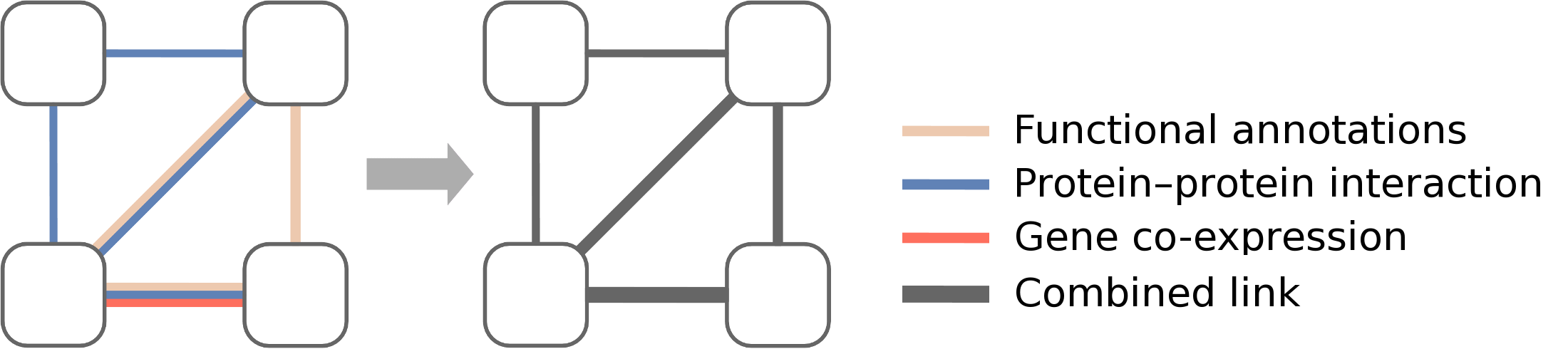

Supplement: Figure S11 — Integration of different data types linking genes. When multiple data sources suggested functional linkage between the same two genes, we integrated the link weights into one for each gene pair. The rounded rectangles represent genes. (PNG) [file pcbi.1003815.s011.png]
