# Supplementary material for: Unbiased Functional Clustering of Gene Variants with a Phenotypic-Linkage Network
Source: PLoS Comput Biol. 2014 Aug 28;10(8):e1003815. doi: 10.1371/journal.pcbi.1003815 (PMC4148192; doi:10.1371/journal.pcbi.1003815)
Supplement: Table S1 — Data sources included in the integrated phenotypic-linkage network. (DOC) [file pcbi.1003815.s012.doc]

**Table S1: Data sources included in the integrated phenotypic-linkage network**

| **Data type** | **Source** | **Form** | **#links** |
| --- | --- | --- | --- |
| Physical interactions | BioGRID, IntAct, Corum, DICS, Reactome | Binary | 493,951 |
| Sequence patterns | InterPro | Semantic similarity | 18,326 |
| Gene expression | GNF2, GSE3594, MTAB-62, Alizadeh *et al*., Kampmann *et al*., Nielsen *et al*., Schaner *et al*., Shyamsundar *et al*. | Correlation coefficient | 1,345,035 |
| Co-citation | STRING* | Scores | 577,887 |
| Pathways, reactions | Reactome, KEGG | Semantic similarity | 71,342 |
| Biological process | Gene Ontology | Semantic similarity | 1,100,459 |
| Molecular function | Gene Ontology | Semantic similarity | 16,379 |
| Cellular location | Gene Ontology | Semantic similarity | 10,757 |

*We used the co-citation of mouse orthologs of human genes in the human gene network.
