# Supplementary material for: Unbiased Functional Clustering of Gene Variants with a Phenotypic-Linkage Network
Source: PLoS Comput Biol. 2014 Aug 28;10(8):e1003815. doi: 10.1371/journal.pcbi.1003815 (PMC4148192; doi:10.1371/journal.pcbi.1003815)
Supplement: Table S2 — Data sources not included in the integrated phenotypic-linkage network. (DOC) [file pcbi.1003815.s013.doc]

**Table S2: Data sources not included in the integrated phenotypic-linkage network**

| **Data type** | **Source** | **Type** |
| --- | --- | --- |
| Physical interactions | IntAct | Mouse proteins MI:0029 cosedimentation through density gradient  Mouse proteins MI:0676 tandem affinity purification |
| Sequence patterns | InterPro | Mouse proteins |
| Gene expression | SMD* | Abate *et al*. 2004  Adler *et al*. 2006  Adler *et al*. 2008  Alizadeh *et al*. 2010  Alter *et al*. 2003  Appari *et al*. 2009  Baldwin *et al*. 2003  Bashyam *et al*. 2005  Bebermeier *et al*. 2006  Beck *et al*. 2010  Ben-Chetrit *et al*. 2006  Benetkiewicz *et al*. 2005  Bergamaschi *et al*. 2006  Bergamaschi *et al*. 2009  Berry *et al*. 2010  Bhamre *et al*. 2009  Blader *et al*. 2001  Bohen *et al*. 2003  Boldrick *et al*. 2002  Bredel *et al*. 2005a  Bredel *et al*. 2005b  Bredel *et al*. 2005c  Bredel *et al*. 2006  Brouard *et al*. 2007  Brown *et al*. 2006  Buess *et al*. 2006  Buess *et al*. 2007  Bullinger *et al*. 2004  Bullinger *et al*. 2007  Bullinger *et al*. 2008  Cario *et al*. 2005  Chang *et al*. 2002  Chang *et al*. 2004  Chang *et al*. 2006  Chen *et al*. 2002  Chen *et al*. 2003  Chen *et al*. 2004  Chi *et al*. 2003a  Chi *et al*. 2003b  Chi *et al*. 2006  Chi *et al*. 2007  Chua *et al*. 2007  Clement *et al*. 2002  Cuadras *et al*. 2002  Dairkee *et al*. 2004  DeAvalos *et al*. 2002  DePrimo *et al*. 2002  Detweiler *et al*. 2001  Diehn *et al*. 2002  Diehn *et al*. 2005  Diehn *et al*. 2006  Dumas *et al*. 2007  El-Etr *et al*. 2004  Faherty *et al*. 2010  Fine *et al*. 2004  Fine *et al*. 2005  Fletcher *et al*. 2009  Fortna *et al*. 2004  Fouts *et al*. 2007  Galgano *et al*. 2008  Garber *et al*. 2001  Giacomini *et al*. 2005  Gilks *et al*. 2005  Griffiths *et al*. 2005  Guillemin *et al*. 2002  Gyorffy *et al*. 2005  Hao *et al*. 2006  Hao *et al*. 2007  He *et al*. 2006  Hendrickson *et al*. 2008  Hendrickson *et al*. 2009  Hertel *et al*. 2004  Heuser *et al*. 2005  Higgins *et al*. 2003  Higgins *et al*. 2004  Higgins *et al*. 2007  Holterhus *et al*. 2003  Holterhus *et al*. 2007  Holweg *et al*. 2011  Houshdaran *et al*. 2010  Hurowitz *et al*. 2007  Iacobuzio *et al*. 2003  Iyer *et al*. 2009  Jacobsen *et al*. 2007  Ji *et al*. 2002  Ji *et al*. 2003  Jones *et al*. 2003  Jones *et al*. 2005a  Jones *et al*. 2005b  Juric *et al*. 2005  Juric *et al*. 2007  Kainz *et al*. 2004  Kao *et al*. 2009  Kapp *et al*. 2006  Kasperkovitz *et al*. 2005  Kharas *et al*. 2010  Kim *et al*. 2007  Klapholz *et al*. 2007  Kosinski *et al*. 2007  Kwei *et al*. 2008a  Kwei *et al*. 2008b  Lacayo *et al*. 2004  Langerod *et al*. 2007  Lapointe *et al*. 2004  Lapointe *et al*. 2007  Lee *et al*. 2006  Lee *et al*. 2008  Leung *et al*. 2002  Leung *et al*. 2004  Li *et al*. 2004  Liang *et al*. 2005  Lin *et al*. 2011  Linn *et al*. 2003  Liu *et al*. 2006  Liu *et al*. 2007  Lossos *et al*. 2002  Lowe *et al*. 2007  Mazzucotelli *et al*. 2007  Melk *et al*. 2005  Mueller *et al*. 2004  Munagala *et al*. 2004  Murray *et al*. 2004  Myers *et al*. 2006  Nagarayan *et al*. 2007  Naume *et al*. 2007  Nicolau *et al*. 2007  Nielsen *et al*. 2004  Novoradovskaya *et al*. 2004  Palmer *et al*. 2006  Pathan *et al*. 2004  Patil *et al*. 2005  Perou *et al*. 1999  Perou *et al*. 2000  Pollack *et al*. 2002  Popper *et al*. 2007  Popper *et al*. 2009  Rajski *et al*. 2010  Rinn *et al*. 2006  Roose *et al*. 2003  Rosenwald *et al*. 2001  Ross *et al*. 2000  Ross *et al*. 2001  Rubins *et al*. 2004  Rubins *et al*. 2007  Rubins *et al*. 2008  Rucker *et al*. 2006a  Rucker *et al*. 2006b  Rudnicki *et al*. 2009  Saaf *et al*. 2006  Saaf *et al*. 2007  Sarwal *et al*. 2003  Shaner *et al*. 2003  Schwarze *et al*. 2002  Segal *et al*. 2007  Simmons *et al*. 2007  Sivertsen *et al*. 2006  Sood *et al*. 2006b  Sood *et al*. 2008  Sorlie *et al*. 2001  Sorlie *et al*. 2003  Sperger *et al*. 2003  Sridhar *et al*. 2009  Subramanian *et al*. 2004  Subramanian *et al*. 2005  Storz *et al*. 2003  Thompson *et al*. 2008  Thompson *et al*. 2009  Timmer *et al*. 2007  Townley-Tilson *et al*. 2006  Tsai *et al*. 2006  VanBaarsen *et al*. 2006  VanBaarsen *et al*. 2008  VanBaarsen *et al*. 2010  VanDerPouw *et al*. 2003a  VanDerPouw *et al*. 2003b  VanDerPouw *et al*. 2007  VanDerPouw *et al*. 2008a  VanDerPouw *et al*. 2008b  Wang *et al*. 2006  Waddel *et al*. 2010  West *et al*. 2004  West *et al*. 2005  West *et al*. 2006  Whitfield *et al*. 2002  Whitfield *et al*. 2003  Whitney *et al*. 2003  Wong *et al*. 2008b  Zhang *et al*. 2003  Zhang *et al*. 2004  Zhang *et al*. 2008  Zhao *et al*. 2002  Zhao *et al*. 2004a  Zhao *et al*. 2004b  Zhao *et al*. 2005a  Zhao *et al*. 2005b  Zhao *et al*. 2005c  Zhao *et al*. 2006 |

*Stanford Microarray Database (http://smd.princeton.edu).
