# Supplementary material for: Unbiased Functional Clustering of Gene Variants with a Phenotypic-Linkage Network
Source: PLoS Comput Biol. 2014 Aug 28;10(8):e1003815. doi: 10.1371/journal.pcbi.1003815 (PMC4148192; doi:10.1371/journal.pcbi.1003815)
Supplement: Table S3 — Fractions of gene pairs co-annotated with the same phenotype in the integrated phenotypic-linkage network. (DOC) [file pcbi.1003815.s014.doc]

**Table S3: Fractions of gene pairs co-annotated with the same phenotype in the integrated phenotypic-linkage network**

| **Gene–gene links*** | **Fraction** | **Phenotype** |
| --- | --- | --- |
| 1 to 10,000 | 5.60%  5.17%  4.47%  3.42%  3.22%  3.10%  3.01%  2.54%  2.51%  2.25% | Abnormality of the nervous system  Abnormality of metabolism/homeostasis  Abnormality of the musculoskeletal system  Abnormality of the eye  Abnormality of the abdomen  Abnormality of the head and neck  Abnormality of the integument  Abnormality of musculature  Abnormality of the genitourinary system  Neoplasia |
| 10,001 to 20,000 | 3.56%  3.44%  3.18%  2.06%  1.98%  1.92%  1.87%  1.81%  1.65%  1.55% | Abnormality of the nervous system  Abnormality of metabolism/homeostasis  Abnormality of the musculoskeletal system  Abnormality of the abdomen  Abnormality of the head and neck  Abnormality of the eye  Abnormality of musculature  Abnormality of the integument  Abnormality of the cardiovascular system  Abnormality of the genitourinary system |
| 20,001 to 30,000 | 3.92%  3.32%  3.18%  2.43%  2.17%  1.95%  1.88%  1.66%  1.63%  1.41% | Abnormality of the nervous system  Abnormality of the musculoskeletal system  Abnormality of metabolism/homeostasis  Abnormality of the eye  Abnormality of the head and neck  Abnormality of the integument  Abnormality of the abdomen  Abnormality of musculature  Abnormality of the genitourinary system  Abnormality of the ear |
| 30,001 to 40,000 | 3.70%  3.19%  2.89%  2.15%  1.93%  1.88%  1.82%  1.63%  1.50%  1.31% | Abnormality of the nervous system  Abnormality of the musculoskeletal system  Abnormality of metabolism/homeostasis  Abnormality of the eye  Abnormality of the head and neck  Abnormality of the abdomen  Abnormality of the integument  Abnormality of musculature  Abnormality of the genitourinary system  Growth abnormality |
| 40,001 to 50,000 | 3.11%  2.72%  2.50%  1.94%  1.80%  1.76%  1.56%  1.33%  1.26%  1.11% | Abnormality of the nervous system  Abnormality of the musculoskeletal system  Abnormality of metabolism/homeostasis  Abnormality of the head and neck  Abnormality of the eye  Abnormality of the integument  Abnormality of the abdomen  Abnormality of musculature  Abnormality of the genitourinary system  Abnormality of the cardiovascular system |

*Ordered by strength.
